# Amyloid *β* 1-42 induces hypometabolism in human stem cell-derived neuron and astrocyte networks

**DOI:** 10.1038/jcbfm.2015.58

**Published:** 2015-04-08

**Authors:** Marta A Tarczyluk, David A Nagel, H Rhein Parri, Erin HY Tse, James E Brown, Michael D Coleman, Eric J Hill

**Affiliations:** 1 Department of Basic and Clinical Neuroscience, James Black Centre, Institute of Psychiatry, London, UK; 2 Aston Research Centre for Healthy Ageing, School of Life and Health Sciences, Aston University, Birmingham, UK

**Keywords:** Alzheimer's disease, amyloid, astrocytes, metabolism, neurons, stem cells

## Abstract

Alzheimer's disease (AD) is the most common form of dementia, affecting more than 35 million people worldwide. Brain hypometabolism is a major feature of AD, appearing decades before cognitive decline and pathologic lesions. To date, the majority of studies on hypometabolism in AD have used transgenic animal models or imaging studies of the human brain. As it is almost impossible to validate these findings using human tissue, alternative models are required. In this study, we show that human stem cell-derived neuron and astrocyte cultures treated with oligomers of amyloid beta 1-42 (A*β*1-42) also display a clear hypometabolism, particularly with regard to utilization of substrates such as glucose, pyruvate, lactate, and glutamate. In addition, a significant increase in the glycogen content of cells was also observed. These changes were accompanied by changes in NAD^+^/NADH, ATP, and glutathione levels, suggesting a disruption in the energy-redox axis within these cultures. The high energy demands associated with neuronal functions such as memory formation and protection from oxidative stress put these cells at particular risk from A*β*-induced hypometabolism. Further research using this model may elucidate the mechanisms associated with A*β*-induced hypometabolism.

## Introduction

It is now widely accepted that Alzheimer's disease (AD) is accompanied by hypometabolism, of differing severity in different regions of the brain. Crucially, signs of hypometbolism such as a reduction in central nervous system glucose utilization as well as mitochondrial function, begin decades before any symptoms or histopathologic changes appear, making such events useful biomarkers of AD risk.^1^ In addition, reductions in key mitochondrial enzyme complex activities such as the *α*-ketoglutarate dehydrogenase complex have been observed.^2^

One obvious explanation for the observed reduction in glucose utilization could be neuronal loss, which is observed in AD patients. However, many studies have shown a reduction in the cerebral metabolic rate of glucose before the onset of the disease and subsequent cell loss. This has been seen in individuals at risk of developing AD such as APOE4 carriers, those carrying autosomal dominant mutations linked with familial AD or patients with mild cognitive impairment.^3^ As hypometabolism is the earliest significant event linked with AD, it suggests that changes in energy metabolism precede neuronal loss and may actually contribute to the development and progression of the disease.

To investigate these changes, a number of *in vivo* studies have attempted to model changes in cerebral glucose utilization in transgenic mice. However, conflicting results have been reported and were dependent on the model used. Studies using models that overexpress the Swedish and Indiana mutations of human amyloid precursor protein^4^ as well as the 3xTG model of AD, which overexpresses human amyloid precursor protein, PS1, and tau mutations^5^ have showed reductions in cerebral glucose uptake. While studies using the Tg2576 mouse model that over expresses the Swedish mutation of amyloid precursor protein,^6^ as well as a model that overexpresses the Swedish and London mutations of amyloid precursor protein, along with PS1^7^ have all displayed an increase in brain glucose uptake.

Furthermore, reductions in glucose uptake in response to A*β* have also been reported *in vitro* using primary rat hippocampal neurons,^8, 9^ while in contrast, an increase in glucose uptake has been reported in primary mouse astrocytes.^10^

Difficulties in obtaining viable human tissue from living patients have made it almost impossible to validate these findings in human neurons. It is clearly important to be able to model glucose metabolic changes in tissues and experimental models that are directly relevant to the disease. While rodent and human brain share a number of common features, rodent models do not naturally develop AD; indeed, it is important note the differences between the human and rodent central nervous system. Human cortical astrocytes have been shown to be both larger and structurally more complex and diverse than those of rodents.^11^ These findings highlight fundamental differences between species used to study human diseases and show the importance of developing functional human models that can be used in comparative studies.

In this way, it may be possible not only to replicate the findings observed in human patients but also to test novel hypotheses that may predict changes in human brain tissue. As such, human stem cell-derived models may provide the most simplistic and relevant platforms to study cell–cell interaction in AD. To this end, we have used human NT2.D1 stem cell-derived neuron and astrocyte co-cultures to determine the effects of exposure to oligomeric A*β*1-42 on cellular metabolism and oxidative stress in comparison with primary rat hippocampal cultures.

The NT2.D1 embryocarcinoma cell line is well characterized and generates neuronal (NT2.N) cells containing heterogeneous subpopulations of dopaminergic, cholinergic, GABAergic, and glutamatergic neurons^12, 13, 14, 15^ as well as astrocytic (NT2.A) cells.^16, 17^ These cells have been shown to generate action potentials on depolarization^18^ and form functional spontaneously active neuronal networks.^19^ We have shown recently that these cells show metabolic coupling and can link neuronal activity to changes in astrocytic metabolism.^20^ Critically, increasing numbers of studies have highlighted the important role of astrocytes in AD.^21^ Indeed, astrocytes in the healthy brain enhance neuronal survival, axonal growth, synaptogenesis, as well as neurovascular and neurometabolic coupling; hence, their inclusion confers structural and functional relevance in any model of brain function.

Crucially, recent studies have also showed the role of human astrocytes early in AD^22^ and have reported metabolic changes after treatment of primary mouse astrocyte cultures with A*β* oligomers^10^ thus supporting the inclusion of astrocytes into *in vitro* models of AD.

As changes in brain metabolism occur decades before any other symptoms, the development of relevant human models of the earliest significant metabolic foundations of the disease may provide important future insights into disease process milestones, that may one day be therapeutic targets. Therefore, this study focused on using cocultures of human NT2-derived neurons and astrocytes to test the hypothesis that A*β* alters cellular metabolism in these cells.

## Materials and methods

### Aggregation Protocol

Hexafluoroisopropanol (HFIP) treated A*β*1-42 (Anaspec, Freemont, CA, USA) was resuspended in 200 mmol/L HEPES, pH 8.5 to a concentration of 100 *μ*mol/L. Treatment with HFIP has previously been shown to dissolve higher aggregates, eliminating the ‘nucleating seeds' and removing any secondary or tertiary structures.^23^ The aliquots were stored at −80°C and used at working concentrations of 20, 2, and 0.2 *μ*mol/L. These concentrations were selected based on the previous research demonstrating the toxicity of Amyloid oligomers in primary cultures.^23, 24^ In addition, these concentrations are within the micromolar concentration range of soluble and insoluble A*β*1-42 levels reported in AD patient brain tissue.^25^

### Cell Culture

Human teratocarcinoma NT2.D1 cells used in this study were kindly donated by Professor Andrews (University of Sheffield, UK). The cells were cultured in DMEM (Dulbecco's Modified Eagle Medium) high glucose, containing, Glutamax, (Life Technologies, Paisley, UK), 10% heat inactivated fetal bovine serum (Life Technologies), 100 units/mL penicillin, and 100 *μ*g/mL streptomycin. NT2.D1 cells were differentiated according to the previously published methods.^26^ Briefly, NT2.D1 cells were differentiated using DMEM Glutamax high glucose medium with pyruvate, supplemented with 10% (v/v) fetal bovine serum, 100 units/mL penicillin, 100 *μ*g/mL streptomycin (pen/strep), and 1 × 10^−5^ M all-trans retinoic acid (RA) (Sigma-Aldrich, Dorset, UK) for 4 weeks. Differentiated cells were seeded at a lower density, then dislodged and re-seeded onto CellBIND 12-well plates (Corning, New York, USA) at 1.25 × 10^6^ cells/well and treated with medium containing antiproliferative agents (MI) for 28 days; 0.1 *μ*mol/L cytosine arabinoside (for the first 7 days only), 3 *μ*mol/L fluorodeoxyuridine, and 5 *μ*mol/L uridine. Pure astrocytic cultures were produced as previously described.^26^ Briefly, NT2.N/A cells were dissociated into a single-cell suspension using Accutase (PAA Laboratories, Yeovil, UK) and subsequently seeded onto fresh CellBIND 12-well plates in MI-free medium. After incubation for 2 hours, the plates were shaken briefly using a plate shaker (400 r.p.m.) to dissociate loose NT2.N cells from the more adherent NT2.A cells leaving an astrocytic mono-layer after rinsing. All cells were maintained by incubation at 37°C in a humidified atmosphere of 5% CO_2_. Unless otherwise stated, all experiments were performed at 37°C in a humidified atmosphere of 5% CO_2_. The proportion of cell types produced by this method in this study were in agreement with previously published values (33±4% neurons and 63±4% astrocytes).^26^

### Primary Cortical Cultures

All procedures were approved by Aston University Bioethics committee, and performed in accordance with the UK Animals (Scientific Procedures) Act 1986 and associated procedures. Cortical cultures were prepared from 2- to 3-day-old male Wistar rats. Animals were anesthetized with isoflurane and euthanized by cervical dislocation. After removal, the brain was placed in ice-cold Gey's salt solution (Sigma-Aldrich) containing 20 *μ*g/mL gentamycin (Life Technologies). The cortex was dissected and placed in ice-cold Gey's salt solution containing 20 *μ*g/mL gentamycin. The tissue was then minced using a scalpel and placed in Ca^2+^-free and Mg^2+^-free Hanks' buffered saline solution (Life Technologies), containing 0.1% trypsin (Life Technologies) for 30 minutes at 37°C. The trypsin was inactivated by adding Neurobasal medium (Life Technologies) containing B27 (Life Technologies), 100 units/mL penicillin and 100 *μ*g/mL streptomycin and 10% horse serum (Life Technologies). Cells were then centrifuged at 258 *g* for 5 minutes and then medium was replaced with 5 mL of fresh Neurobasal medium. Cells were dissociated by trituration with a glass Pasteur pipette with a flame-rounded tip and passed through 70 *μ*m filter (BD Biosciences, Oxford, UK). Cells were counted using a hemocytometer and plated onto poly-D-lysine coated 12-well plates at a final concentration of 5 × 10^5^ cells/mL. Cells were maintained at 37°C and 5% CO_2_ and fed twice a week, cells were used after 5 days. Plates for primary cortical cultures were coated with poly-D-lysine (Sigma-Aldrich) at a concentration of 50 *μ*g/mL. Briefly, poly-D-lysine was resuspended in sterile H_2_O and filtered through 0.22 *μ*m filter, the wells were coated with 2 mL of the solution and incubated at 37°C overnight. The poly-D-lysine was aspirated and plates were rinsed with sterile H_2_O and dried.

### Cell Viability

The viability of the cultures after treatment with A*β*1-42 was determined using the Cell-titre Blue assay (Promega, Southampton, UK). After experimental treatment, medium was removed from the wells of the 12-well cell-culture plate. Subsequently, the plate was washed with 500 *μ*L phenol red-free DMEM media (Life Technologies), supplemented with 10% heat inactivated fetal bovine serum (NT2.N/A, NT2.A) or 10% horse serum (primary cortical cultures), 100 units/mL penicillin and 100 *μ*g/mL streptomycin and 2 mmol/L L-glutamine. In all, 1 mL of Cell-titre blue reagent was mixed with 10 mL of the DMEM. In all, 500 *μ*L of this solution was added to each well of the plate. The plate was incubated for 3 hours at 37°C. After incubation, medium was transferred to a 96-well plate and the absorbance was measured at 590 nm using a Thermo multiscan EX 96-well plate reader (Thermofisher, Loughborough, UK).

### Determination of Carbohydrate Levels

The levels of glycogen in biological samples were determined using the method described by Nahorski and Rogers.^27^ Lactate was measured using the Fluorescent Lactate Assay Kit (Abcam, Cambridge, UK) according to the manufacturer's instructions. Pyruvate was measured using the Pyruvate assay kit (Abcam) according to the manufacturer's instructions. Glucose levels were measured using Glucose (HK) Assay Kit (Sigma-Aldrich) according to the manufacturer's instructions.

### NAD/NADH

NAD^+^/NADH ratio was measured using the NAD/NADH assay kit (Abcam) according to the manufacturer's instructions.

### ATP

ATP levels were measured using the CellTiter-Glo kit (Promega) according to the manufacturer's instructions.

### Determination of Glutamate Levels

Glutamate levels were measured using Amplex Red Glutamic Acid/Glutamate Oxidase Assay kit (Life Technologies).

### Glutathione (GSH)/Glutathione Disulfide (GSSG) Assay

Glutathione/GSSG was measured as previously described.^28^

### Statistics

Results were expressed as the mean of three samples±standard error of the mean (s.e.m.). Comparisons between treatments were performed using analysis of variance (ANOVA) followed by Dunnett's or Tukey's post test or where appropriate Student's *T*-test using GraphPad Prism Software (GraphPad Prism software, La Jolla, CA, USA). Differences were considered as significant for *P* values <0.05.

## Results

### Characterization of Cells

**Figure 1:**
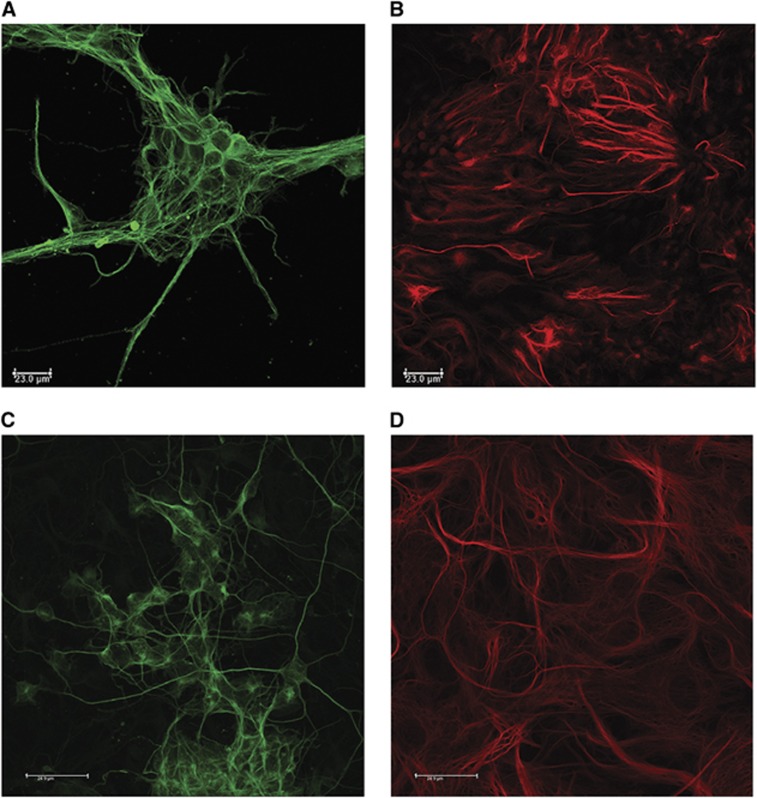
Immunofluorescent images of NT2/D1-derived neurons and astrocytes (**A** and **B**) and primary cortical mixed neuronal and glial cultures (**C** and **D**). Images showing (**A**) *β*-tubulin-positive neurons (green) and (**B**) GFAP- (red) positive astrocytes. Scale bar 23 *μ*m. Images showing (**C**) *β*-tubulin-positive neurons (green) and (**D**) GFAP- (red) positive astrocytes. Scale bar 24.9 *μ*m.

After differentiation, the cultures began to display distinct neuronal and astrocytic morphology. Neurons extended axons and dendrites, and astrocytes with projections appeared in close proximity to aggregations of neuronal perikarya and neurites throughout the culture. Under the microscope astrocytes were identified by their flat phase dark appearance, while neurons were typically phase bright and often seen on top of the astrocytic monolayer. Identification was confirmed using immunohistochemistry for the specific markers GFAP and *β*-tubulin (Figures 1A and 1B).

Primary mixed glial and neuronal cultures were prepared from cortices of Wistar rat pups and maintained in Neurobasal media. These cultures produced a mixed culture of astrocytes and neurons (Figures 1C and 1D).

### Amyloid Aggregation

**Figure 2:**
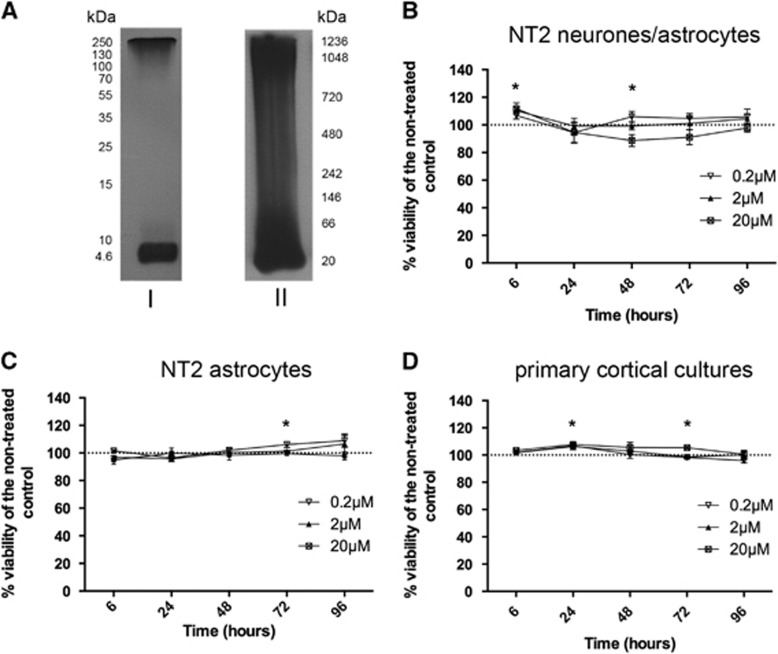
(**A**) Representative western blot analysis of Amyloid Beta 1-42 (A*β*(1-42)) dissolved in HEPES and separated using (I) denaturing and (II) native conditions. Viability results after treatment with 20, 2, and 0.2 *μ*mol/L A*β*. (**B**) NT2.N/A, (**C**) NT2.A, and (**D**) primary cortical cultures. Viability was measured after 6, 24, 48, 72, and 96 hours. Results are expressed as percentage of nontreated control±s.e.m., *n*=3. *P*<0.05 (*). Comparisons between treatments were performed using analysis of variance (ANOVA) followed by Dunnet's post test.

To assess the composition of each preparation, freshly prepared samples of A*β*(1-42) at a concentration of 20 *μ*mol/L were separated using SDS and Native polyacrylamide gel electrophoresis and visualized using western blotting (Figure 2A). A*β*(1-42) prepared in 100 mmol/L HEPES at pH 8.5 showed a large proportion of monomers at around ~4 kDa and high n-oligomers at the top of the gel (~130 to 250 kDa) (Figure 2A).

### The Effects of Amyloid on Cell Viability

To determine the temporal and concentration-dependent effects of A*β* on NT2.N/A cocultures, NT2.A cultures, and mixed rat glial and neuronal cultures, cells were treated with different concentrations of A*β*1-42 oligomers (0.2, 2, and 20 *μ*mol/L) for 6, 24, 48, 72, and 96 hours. After treatment, the viability of the cells was measured indirectly using Cell-titre blue assay (Promega).

In cocultures the only significant change was seen at the highest concentration of A*β*1-42 when compared with the untreated control (Figure 2B). Application of 20 *μ*mol/L A*β* caused an increase in reduction of resazurin at 6 hours (112±4.2%, *P*<0.05) and a decrease at 48 hours (89±4.3%, *P*<0.05) compared with control. In the pure astrocytic cultures, there was a significant increase in the reduction of resazurin in the cells treated with 0.2 *μ*mol/L A*β* for 72 hours (106±2.1%, *P*<0.05) (Figure 2C). Similarly the primary cortical cultures did not show any significant cell death over time (Figure 2D). After 24 hours treatment with 20 and 0.2 *μ*mol/L the cultures showed an increase in the reduction of resazurin (20 *μ*mol/L: 107±1.7, *P*<0.05; 0.2 *μ*mol/L: 108±1.3%, *P*<0.05). The same increase in the reduction of resazurin was seen after 72 hours treatment with the highest concentration of A*β* (106±0.99%, *P*<0.05).

### Glucose Uptake Is Decreased After Treatment with Amyloid Beta

**Figure 3:**
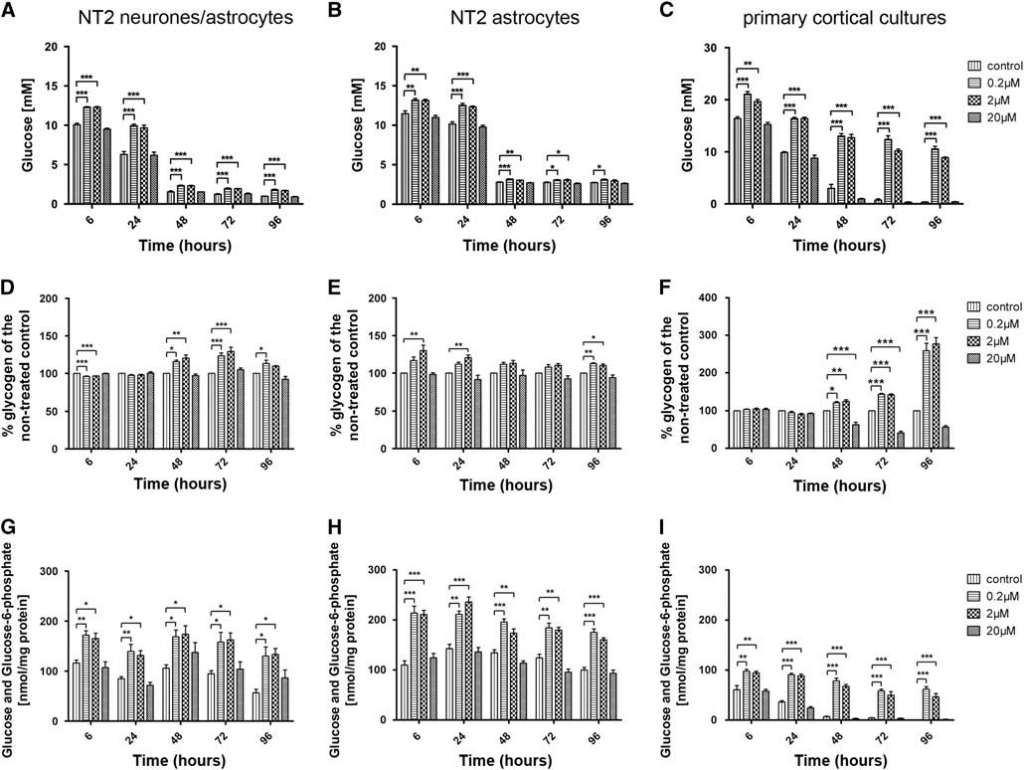
Glucose levels in the media and glycogen and glucose/glucose phosphate levels inside the cells after treatment of cultures with 20 , 2, and 0.2 *μ*mol/L amyloid beta (A*β*). Metabolite levels were measured after 6, 24, 48, 72, and 96 hours. Glucose levels in the media. (**A**) NT2.N/A, (**B**) NT2.A, and (**C**) primary cortical cultures. Results are expressed as mmol/L±s.e.m., *n*=3. Glycogen levels inside the cells (**D**) NT2.N/A, (**E**) NT2.A, and (**F**) primary cortical cultures. Glucose and Glucose-6-phosphate levels inside the cells (**G**) NT2.N/A, (**H**) NT2.A, and (**I**) primary cortical cultures. Results are expressed as nmol/mg protein±s.e.m., *n*=3 *P*<0.05 (*), *P*<0.01 (**), *P*<0.001 (***). Comparisons between treatments were performed using analysis of variance (ANOVA) followed by Dunnet's post test.

To determine the effect of amyloid on glucose uptake, the glucose concentration of media from A*β*-treated cells was measured using the Glucose (Hexokinase) Assay Kit (Sigma-Aldrich). NT2.N/A, NT2.A and primary cortical cultures all showed a significant decrease in glucose uptake after treatment with 2  and 0.2 *μ*mol/L A*β* at all time points investigated. Glucose levels in the medium from cocultures were significantly increased (*P*<0.001) at all time points (Figure 3A). Similar increases in the glucose content of culture media were also seen in primary cortical cultures (*P*<0.001) at all time points with the exception of 2 *μ*mol/L A*β* treatment at 6 hours (Figure 3C). Astrocytic cultures also showed a decrease in glucose uptake, though to a lesser extent than neuronal and astrocytic cocultures (Figure 3B). At 24 hours, the glucose levels in the media were significantly increased after the treatment with A*β* (control: 10.2±0.29 mmol/L; 2 *μ*mol/L: 12.3±0.09 mmol/L, *P*<0.001; 0.2 *μ*mol/L: 12.5±0.25 mmol/L, *P*<0.001). The decrease in glucose uptake became less significant over time in astrocytes. At 72 hours, the increase in glucose content in culture media is at *P*<0.05 for both concentrations and at 96 hours only 0.2 *μ*mol/L A*β* treatment had any substantial impact (*P*<0.05).

The glucose uptake over time differed between the cultures. In all cases the starting concentration of glucose in the medium was 25 mmol/L. The NT2.N/A cultures used up over 50% of the available glucose in the first 6 hours (control: 10.1±0.19 mmol/L). Pure astrocytic cultures took up less glucose than cocultures (control: 11.5±0.36 mmol/L; *P*<0.05) while uptake in primary cultures was even slower (control: 16.4±0.31 mmol/L; *P*<0.0001).

Primary cortical cultures were more sensitive to A*β* treatment and glucose uptake was blocked to a greater extent than in NT2.N/A or NT2.A cultures. After 96 hours, primary cortical cultures treated with 0.2 *μ*mol/L A*β* depleted 57.7±2%, (*P*<0.001) of the glucose from the media compared with the control (98.6±0.25%) while NT2.N/A used up 92.8±0.28%, compared with the control (96.1±0.05% *P*<0.001 ) and NT2.A 87.5±0.34%, compared with the control (89.1±0.17% *P*<0.05).

### Intracellular Glucose and Glucose-6-Phosphate Is Increased After Treatment with Amyloid Beta

Intracellular glucose and glucose-6-phosphate levels were measured after the treatment with A*β*. As in previous experiments the changes in glucose levels were restricted to the two lower concentrations of A*β*, 2  and 0.2 *μ*mol/L. The NT2.N/A cocultures showed an accumulation of glucose and glucose-6-phosphate at all time points (Figure 3G). The increase in glucose content was most significant at 6 hours (control: 116±5.61 nmol/mg protein; 0.2 *μ*mol/L: 172±9.06 nmol/mg protein; *P*<0.01) and 24 hours time point (control: 84.8±3.9 nmol/mg protein; 0.2 *μ*mol/L: 139±14.1 nmol/mg protein; *P*<0.01). A more significant accumulation of glucose and glucose-6-phosphate was observed in astrocytes than in cocultures, with 6 hours (control: 110±8.4 nmol/mg protein; 0.2 *μ*mol/L: 214±14 nmol/mg protein, *P*<0.001) and 24 hours time points (control: 143±8.6 nmol/mg protein; 0.2 *μ*mol/L: 211±6.3 nmol/mg protein, *P*<0.001) showing the largest increase (Figure 3H). Similarly primary cortical cultures also showed a very significant (*P*<0.001) accumulation at all time points after treatment with A*β* (Figure 3I).

### Intracellular Glycogen Is Increased after Treatment with Amyloid Beta

The levels of glycogen within the control cultures were stable throughout the incubation period (Figure 3D). After the treatment of the cultures with A*β*, glycogen levels inside the cells were measured at 6, 24, 48, 72, and 96 hours. NT2.N/A cocultures showed an initial decrease in glycogen levels at 6 hours compared with control (0.2 *μ*mol/L: 96.1±0.47%, *P*<0.001; 2 *μ*mol/L: 96.6±0.15%, *P*<0.001) (Figure 3D). Glycogen levels increased to control levels at 24 hours, with significant (*P*<0.001) increases at 72 hours to 129±5.57% when treated with 2 *μ*mol/L A*β* and to 123±3.94% with 0.2 *μ*mol/L A*β*. At 96 hours, the differences were less apparent with only 0.2 *μ*mol/L A*β* having a significant effect (113±4.32%, *P*<0.05) (Figure 3D). Primary cortical cultures showed a similar pattern, with glycogen levels increasing at 48 hours. However, the increase in glycogen was more significant and at 96 hours levels of glycogen reached 277±16.5% (*P*<0.001) after treatment with 2 *μ*mol/L A*β* and 259±19.9% (*P*<0.001) when treated with 0.2 *μ*mol/L A*β* (Figure 3F). Additionally, from 48 hours there was a decrease in glycogen levels in cells treated with 20 *μ*mol/L A*β* which was significant at 48 hours (*P*<0.001) and 72 hours (*P*<0.001).

Pure astrocytes also showed an increase in glycogen levels from 6 hours (2 *μ*mol/L: 130±7.1%, *P*<0.01) (Figure 3E). Increases were observed at all time points, but were only significant at 24 and 96 hours.

### Lactate Levels in the Cell Conditioned Media Are Significantly Decreased After Treatment with Amyloid Beta

Lactate levels in the cell conditioned media after treatment with A*β* were found to be significantly decreased in NT2.N/A cocultures. At 6 hours, there was no significant change in lactate levels.

**Figure 4:**
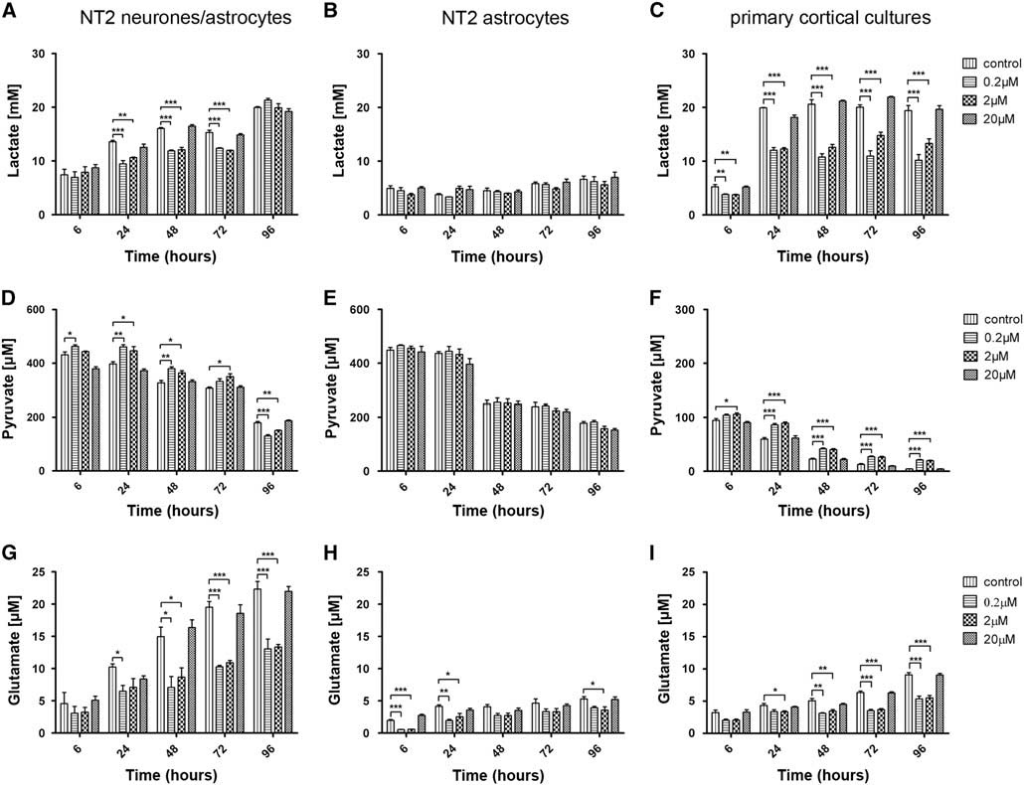
Lactate, pyruvate, and glutamate levels in the media after treatment of cultures with 20, 2, and 0.2 *μ*mol/L Amyloid beta (A*β*). Metabolite levels were measured after 6, 24, 48, 72, and 96 hours. Lactate levels in the media (**A**) NT2.N/A, (**B**) NT2.A, and (**C**) primary cortical cultures. Results are expressed as mmol/L±s.e.m., *n*=3. Pyruvate levels in the media (**D**) NT2.N/A, (**E**) NT2.A, and (**F**) primary cortical cultures. Results are expressed as mmol/L±s.e.m., *n*=3. Glutamate levels in the media (**G**) NT2.N/A, (**H**) NT2.A, and (**I**) primary cortical cultures. Results are expressed as *μ*mol/L±s.e.m., *n*=3. *P*<0.05 (*), *P*<0.01 (**), *P*<0.001 (***). Comparisons between treatments were performed using analysis of variance (ANOVA) followed by Dunnet's post test.

However, after 24 hours the levels were decreased after treatment with both 0.2  and 2 *μ*mol/L A*β* (control: 13.6±0.3 mmol/L; 2 *μ*mol/L: 10.6±0.1 mmol/L, *P*<0.01; 0.2 *μ*mol/L: 9.5±0.6 mmol/L, *P*<0.001) (Figure 4A). The decrease was also seen at 48 and 72 hours; however, at 96 hours, levels of lactate were similar to control levels (control: 19.95±0.18 mmol/L; 2 *μ*mol/L: 19.9±0.78 mmol/L; 0.2 *μ*mol/L: 21.3±0.33 mmol/L). Pure astrocytes did not show any change after the treatment with A*β*. Interestingly, levels of lactate in pure astrocyte cultures were much lower than in cocultures (Figure 4B). In primary cortical cultures, the decrease in lactate was very significant and was seen from 6 hours (control: 5.23±0.41 mmol/L; 2 *μ*mol/L: 3.75±0.10 mmol/L, *P*<0.01; 0.2 *μ*mol/L: 3.8±0.1 mmol/L, *P*<0.01) through to 96 hours (control: 19.4±0.97 mmol/L; 2 *μ*mol/L: 13.3±0.9 mmol/L, *P*<0.001; 0.2 *μ*mol/L: 10.2±1.1 mmol/L, *P*<0.001) (Figure 4C).

Lactate was found to accumulate over time in all cultures. In NT2.N/A cultures and primary cultures, the lactate levels were much higher than in pure astrocytes. Additionally, in NT2.N/A cocultures the accumulation of lactate was slower than in primary cultures. NT2.N/A lactate reached 19.95±0.18 mmol/L levels at 96 hours while primary cultures attained 20.6±0.8 mmol/L levels at 48 hours after which time the levels remained stable.

### Pyruvate Levels in the Cell Conditioned Media Are Significantly Increased After Treatment with Amyloid Beta

The levels of pyruvate in the cell culture media were measured after treatment with A*β*. The initial pyruvate concentration in NT2.N/A and NT2.A medium was 1 mmol/L while in primary cortical cultures the initial pyruvate concentration was 200 *μ*mol/L.

All cultures showed a significant reduction in pyruvate levels in cell culture media over time regardless of treatment. In NT2.N/A cultures treated with both 0.2 and 2 *μ*mol/L A*β*, the pyruvate content in the cell culture media was higher than the control. The most significant increase was seen at 24 hours (control: 398±7.7 *μ*mol/L; 2 *μ*mol/L: 447±15.9 *μ*mol/L, *P*<0.05; 0.2 *μ*mol/L: 461±8.3 *μ*mol/L, *P*<0.01) and 48 hours (control: 327±8.7 *μ*mol/L; 2 *μ*mol/L: 364±7.6 *μ*mol/L, *P*<0.05; 0.2 *μ*mol/L: 380±6.97 *μ*mol/L, *P*<0.01) (Figure 4D). However, at 96 hours the level of pyruvate decreased significantly in comparison with control (Figure 4D). In pure astrocytes, there was no change in the concentration of pyruvate after the treatment with A*β* (Figure 4E). Primary cortical cultures showed a similar pattern to NT2.N/A cocultures, with an increase in the concentration of pyruvate becoming more significant after 6 hours (Figure 4F). Conversely to NT2.N/A cocultures (Figure 4F) there was no decrease at 96 hours in primary cortical culture (control: 4.19±0.44 *μ*mol/L; 2 *μ*mol/L: 19.6±0.89 *μ*mol/L, *P*<0.001; 0.2 *μ*mol/L: 21.0±0.91 *μ*mol/L, *P*<0.001).

### Glutamate Levels in the Cell Conditioned Media Are Significantly Decreased after Treatment with Amyloid Beta

Treatment with A*β* was also found to have an effect on glutamate levels in the media. In NT2.N/A cocultures, there was a significant decrease in glutamate levels from 24 hours with the largest decrease at 72 hours and 96 hours (control: 22.3±1.19 *μ*mol/L; 2 *μ*mol/L: 13.3±0.41 *μ*mol/L, *P*<0.001; 0.2 *μ*mol/L: 13.05±1.52 *μ*mol/L, *P*<0.001) (Figure 4G). This decrease became more significant with time, as the control and 20 *μ*mol/L A*β-*treated cells accumulated more glutamate in the media. In pure astrocytes, the decrease was also present but to a lesser extent (Figure 4H). Overall, the levels of glutamate in the media samples were markedly lower in pure astrocytic cultures than cocultures of neurons and astrocytes. Primary cortical cultures also showed a significant decrease in glutamate levels after treatment with 2 and 0.2 *μ*mol/L A*β*. The decrease was significant from 24 hours and most significant at 96 hours (control: 9.08±0.34 *μ*mol/L; 2 *μ*mol/L: 5.51±0.37 *μ*mol/L, *P*<0.001; 0.2 *μ*mol/L: 5.35±0.43 *μ*mol/L, *P*<0.001) (Figure 4I).

### The Cellular GSH/GSSG Ratio Is Significantly Decreased after Treatment with Amyloid Beta

**Figure 5:**
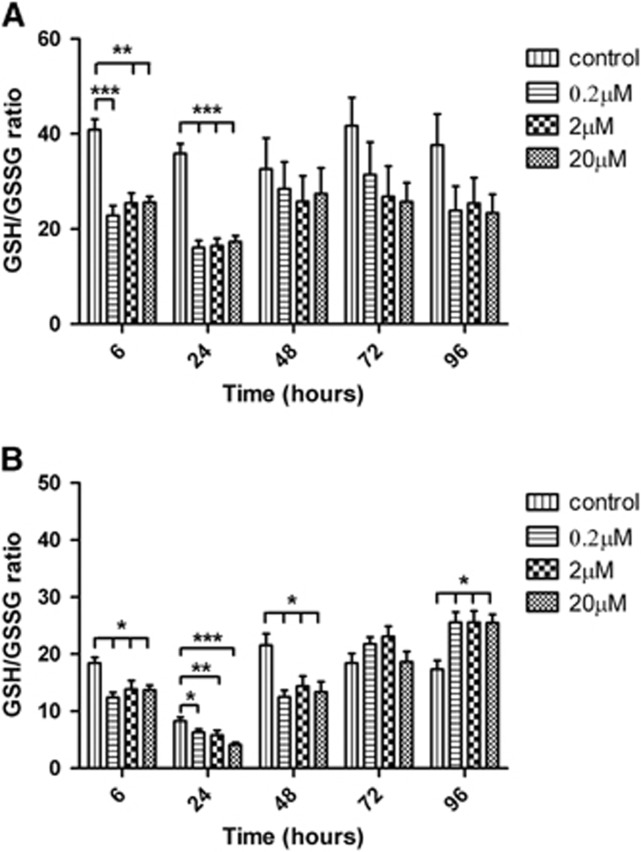
GSH/GSSG ratio inside the cells after treatment with 20, 2, and 0.2 *μ*mol/L Amyloid beta (A*β*). (**A**) NT2.N/A and (**B**) NT2.A. GSH and GSSG were measured after 6, 24, 48, 72, and 96 hours and were normalized to protein concentration. Results are expressed as ratio±s.e.m., *n*=3. *P*<0.05 (*), *P*<0.01 (**), *P*<0.001 (***). Comparisons between treatments were performed using analysis of variance (ANOVA) followed by Dunnet's post test.

The total GSH and GSSG levels of cultures varied dependent on the length of incubation and treatment (Supplementary material). NT2.N/A cocultures treated with all concentrations of A*β* showed a significant decrease in the GSH/GSSG ratio at 6 and 24 hours (Figure 5A). Pure astrocytes also showed a decrease in GSH/GSSG ratio from the 6-hour time point (Figure 5B). This decrease was significant up to 48 hours time point. After 72 hours, the GSH/GSSG ratio increased to control ratio (control: 18.4±1.67; 20 *μ*mol/L: 18.7±1.8; 2 *μ*mol/L: 23.1±1.8; 0.2 *μ*mol/L: 21.8±1.22). At 96 hours, there was a further increase in the ratio (Figure 5B), which was significant at all three concentrations (*P*<0.05). In NT2.N/A after treatment with A*β* GSSG levels varied from 2.74% to 5.71% of the total GSH-GSSG levels, compared with the controls 1.75% to 2.89%. In NT2.A after treatments GSSG levels were higher varying from 3.67% to 18.2%, with controls 4.76% to 10.7%.

### The Cellular NAD+/NADH Ratios and ATP Levels Are Significantly Decreased after Treatment with Amyloid Beta

**Figure 6:**
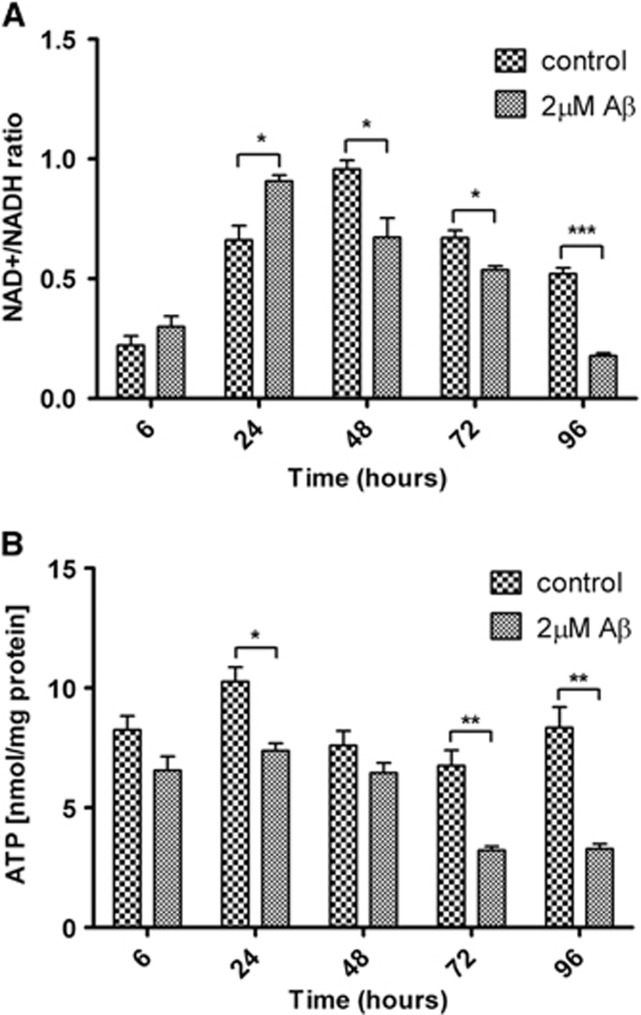
NAD^+^/NADH ratio, and ATP levels inside the cells after treatment of NT2.N/A with 2 *μ*mol/L amyloid beta (A*β*). (**A**) NAD+, NADH, and (**B**) ATP were measured after 6, 24, 48, 72, and 96 hours. Results are expressed as ratio±s.e.m. (**A**) and nmol/mg protein±s.e.m. (**B**), *n*=3. *P*<0.05 (*), *P*<0.01 (**), *P*<0.001 (***). Comparisons between treatments were performed using Students *T*-test.

NAD^+^/NADH ratios and ATP levels were measured in NT2.N/A cocultures after treatment with 2 *μ*mol/L A*β*. The NAD^+^/NADH ratio increased at 24 hours and was followed by a steady decrease at 48, 72, and 96 hours (Figure 6A). Both A*β* treated and control cells followed this trend. There was an initial increase in NAD^+^/NADH ratio after the treatment with A*β* at 24 hours (control: 0.66±0.06; 2 *μ*mol/L: 0.91±0.02, *P*<0.05). However, after this time point the ratio was much lower after the A*β* treatment and became significantly lower at 96 hours (control: 0.52±0.03; 2 *μ*mol/L: 0.18±0.01, *P*<0.001). At 96 hours NAD^+^ levels in control were 820±17.5 pmol/mg of protein, while after treatment with 2 *μ*mol/L, A*β* levels decreased to 348±20.2 pmol/mg of protein. ATP levels after treatment with 2 *μ*mol/L A*β* decreased from 6 hours, becoming significant at 24 hours (Figure 6B). ATP levels reached their lowest levels at 72 and 96 hours (control: 8.34±0.86 nmol/mg protein; 2 *μ*mol/L: 3.29±0.20 nmol/mg protein, *P*<0.01).

## Discussion

Difficulties in studying the cellular mechanisms of AD in living patients combined with shortcomings in the relevance of animal models have confined much AD research to the end-stage pathologies (A*β* plaques and Tau tangles). Clinical imaging approaches in mild cognitive impairment or early-stage AD have provided intriguing insights into the early stages of AD but have been unable to explore these changes at the cellular level.

In this study, we provide the first description of the impact of A*β* on glucose homeostasis in neurons and astrocytes derived from human stem cells. Furthermore, we show changes in the utilization of metabolites such as lactate, pyruvate, and glutamate. In addition, we show an increase in the storage of glycogen as well as changes in the NAD/NADH ratio as well as ATP levels within cells. Since hypometabolism is a key feature of AD, this model may provide a complex functional *in vitro* system for studying early changes in disease pathology.

Human stem cell-derived cocultures of neurons and astrocytes successfully modelled the earliest metabolic changes induced by A*β*. In addition, results obtained from these cells were compared with a well-characterized primary rat cortical tissue culture preparation. As the NT2 line is a form of human stem cell, our protocol concept in this report should inform the design of future studies using patient-derived induced pluripotent stem cells. NT2 cultures treated acutely with exogenous A*β* in this study show ‘metabolic toxicity' over a period of hours/days. As such changes in metabolic homeostasis are observed before the development of symptoms in patients, it would be of great interest to model the long-term effects of A*β* on cellular viability in culture. However, caution should be taken with the interpretation of this response when modelling a disease that may take years/decades to develop *in vivo*. In addition, the concentration of glucose in the media used (25 mmol/L) may impact upon the metabolism of cells and therefore their survival after A*β* treatment. Future studies will determine the effect of low glucose concentrations on cell survival.

After exposure to A*β*, the cultures showed a decrease in the uptake and/or utilization of glucose. A decrease in glucose uptake upon treatment with 2  and 0.2 *μ*mol/L A*β*1-42 could be explained by the accumulation of glucose and glucose-6-phosphate that was observed intracellularly. This could lead to a reduction in the flow of glucose through the hexokinase pathway due to allosteric feedback inhibition of hexokinase. Indeed amyloid itself may decrease hexokinase activity via interference with hexokinase activity and subcellular localization.^29^

The increase in glycogen levels after the treatment with 2 and 0.2 *μ*mol/L A*β*1-42 could also be explained by accumulation of glucose-6-phosphate, which instead of passing through glycolysis, is directed through glycogen synthesis pathways. However, very little is understood about the storage and metabolism of glycogen during AD and further research is required to understand the impacts of these changes on cellular processes.

In this report, glutamate production in both rat cortical and human NT2.N/A cocultures was shown to be affected after treatment with A*β*1-42. In addition, overall glutamate levels in pure NT2-A astrocytic cultures were lower compared with those of cocultures. This is not unexpected as the majority of glutamate is produced by neurons, while astrocytes take up the glutamate released by neurons and convert it to glutamine. Previous studies of AD patient brains have showed a decrease in glutamine synthase, as well as a marked decrease in phosphate-activated glutaminase levels and α-ketoglutarate dehydrogenase complex.^2, 30, 31, 32, 33^ Such changes could conceivably lead to disturbances in glutamate production and recycling *in vivo*. Indeed, studies using magnetic resonance spectroscopy have showed that patients with AD have lower levels of brain glutamate compared to controls with mild cognitive impairmen patients demonstrating intermediate levels.^34^

After exposure to A*β*1-42, lactate levels were significantly decreased in both coculture systems. Interestingly, this effect is not seen in pure astrocytes. The results shown herein strongly suggest that A*β*1-42 leads to inhibition of the glycolytic pathway, which results in the accumulation of glucose/glucose-6-phosphate, decreased uptake of glucose and therefore possibly lactate production. The pathway involved in the production of lactate by astrocytes in the brain is unclear. According to the Astrocyte Neuron Lactate Shuttle hypothesis, lactate production in astrocytes is triggered in response to glutamate uptake.^35^ However, Dienel and McKenna^36^ have suggested various other metabolic pathways including glutamate oxidation and glycolysis with lactate release that can contribute to the energy demands of excitatory neurotransmission.

Uptake of pyruvate, which has been shown to be an important energy source in isolated mitochondria from brain,^37^ was also decreased after treatment with low levels of A*β*1-42 in NT2.N/A cocultures and primary cortical cultures, further limiting availability of substrates for the TCA cycle. However, the relevance of this finding to the whole brain is unclear with brain cells relying almost exclusively on glucose to meet its energy demands.

Interestingly, higher concentrations of amyloid (20 *μ*mol/L) did not have the same effects on metabolism as lower concentrations (2 and 0.2 *μ*mol/L). The kinetics of protofibril formation are greatly dependent on ambient conditions such as temperature, buffer composition, and peptide concentration.^38^ It is possible that higher concentrations of amyloid used in this study may aggregate faster and thus produce amyloid species with altered toxic effects. As such, it is conceivable that such species may not initiate the metabolic effects observed in these cultures after treatment with lower concentrations.

As the treatment of cells with A*β*1-42 leads to a decrease in the substrates available for entry into the TCA cycle, changes in the ratio of NAD^+^/NADH were investigated. Cocultures showed an initial increase in NAD^+^/NADH ratio in both control and A*β*-treated cells and this was followed by a significant decrease, particularly in A*β*-treated cells. Increases in the NAD^+^/NADH ratio seen in the control cultures is possibly a result of increased glycolysis and subsequent production of lactate after the application of fresh media.

During glycolysis NAD^+^ is reduced to NADH. Normally regeneration of NAD^+^ takes place in mitochondria via the malate/aspartate shuttle. However, it has been shown that astrocytes express low levels of a key component of this shuttle and may instead use the conversion of pyruvate to lactate by lactate dehydrogenase to regenerate NAD.^39, 40^

A decrease in the availability of substrates (glucose, pyruvate) and an increase in the requirement for NAD^+^ can lead to NAD^+^ depletion. In addition, decrease in NAD^+^ stores leads to a heavy demand on ATP stores for synthesis of new NAD^+^,^41^ which in turn could provoke an energy crisis in the cell.^42^ In this study, treatment of NT2.D1-derived cocultures with 0.2 *μ*mol/L A*β*1-42 caused a significant decrease in ATP levels from 6 hours onwards in comparison with the control, further highlighting the negative impact of the peptide on cellular energy metabolism.

Dringen *et al*^43^ have showed that detoxification of peroxide by neurons is less efficient than astrocytes, indicating an increase in the susceptibility of neurons to oxidative stress compared with astrocytes. As oxidative stress has been associated with AD, NT2.N/A and NT2.A cultures were investigated for increases in GSH oxidation in response to oxidative stress after treatment with A*β*1-42. Pure astrocytic cultures showed a decrease in GSH/GSSG ratio. After 48 hours, the ratio increased suggesting a recovery from the initial insult. A similar decrease in the GSH/GSSG ratio was seen in cocultures at 6 and 24 hours. A study by Abramov *et al*^44^ performed on rat mixed hippocampal neuronal and glial cultures as well as on pure cortical astrocytes has linked sporadic Ca^2+^ signals caused by A*β* to GSH depletion. Such Ca^2+^ fluctuations were only seen in astrocytes and were associated with a decrease in GSH in those cells. Astrocytes in these cultures were found to withstand this insult and were resistant to cell death, while neurons were found to die within 24 hours after exposure, highlighting the importance of cocultures.^44^ Results described by Dringen *et al*^43^ and Abramov *et al*^44^ also suggest that a compromised astrocytic glutathione system may contribute to a lower defence capacity of the brain against reactive oxygen species. As hypoglycaemic conditions may deplete NADPH levels, this may in turn increase cells susceptibility to oxidative damage due to a limited ability to reduce GSSG to GSH.

As numerous cell types interact during the progression of AD, it is important that any *in vitro* model of the disease reflects this arrangement. Data presented here provide evidence for a detrimental effect of A*β* on carbohydrate metabolism in both neurons and astrocytes. Replication of results using a well-established primary cortical culture model system shows the utility of stem cell-derived neurons and astrocytes for studying hypometabolism observed in AD. As a purely *in vitro* system, human stem cell models can be readily manipulated and maintained in culture for a period of months without the use of animals. In our laboratory, the NT2 model can be maintained in culture for over 6 months thus providing the opportunity to study the consequences of these changes over extended periods of time relevant to aspects of the disease progression timeframe *in vivo*. In addition, their human origin provides a more realistic *in vitro* model as well as informing other human *in vitro* models such as patient-derived induced pluripotent stem cells. Future studies will attempt to investigate the earliest cell-specific changes that lead to hypometabolism using this model as well as the effects of abnormal metabolism on neuronal and astrocytic activity.
