# Supplementary material for: Amyloid β 1-42 induces hypometabolism in human stem cell-derived neuron and astrocyte networks
Source: J Cereb Blood Flow Metab. 2015 Apr 8;35(8):1348–57. doi: 10.1038/jcbfm.2015.58 (PMC4528011; doi:10.1038/jcbfm.2015.58)
Supplement: Supplementary Information [file jcbfm201558x1.doc]

Table 1. Absolute values for GSH [nmol/mg of protein] in NT2.N/A cultures following treatment with 20μM, 2μM, 0.2μM Aβ.

|  | Aβ1-42 concentration (μM) | | | |
| --- | --- | --- | --- | --- |
| Time (hours) | 20 | 2 | 0.2 | control |
| 6 | 10.83 ± 0.22 | 11.85 ± 0.13 | 11.46 ± 0.07 | 13.27 ± 0.24 |
| 24 | 11.56 ± 0.27 | 11.06 ± 0.31 | 10.86 ± 0.47 | 13.60 ± 0.21 |
| 48 | 15.14 ± 5.42 | 12.54 ± 3.65 | 14.00 ± 5.88 | 11.09 ± 2.48 |
| 72 | 15.74 ± 2.48 | 23.03 ± 6.86 | 19.35 ± 3.97 | 20.52 ± 6.83 |
| 96 | 14.50 ± 2.96 | 16.26 ± 3.91 | 14.85 ± 3.15 | 15.67 ± 3.75 |

Table 2. Absolute values for GSSG [nmol/mg of protein] in NT2.N/A cultures following treatment with 20μM, 2μM, 0.2μM Aβ.

|  | Aβ1-42 concentration (μM) | | | |
| --- | --- | --- | --- | --- |
| Time (hours) | 20 | 2 | 0.2 | control |
| 6 | 0.44 ± 0.02 | 0.45 ± 0.03 | 0.47 ± 0.03 | 0.32 ± 0.01 |
| 24 | 0.61 ± 0.03 | 0.63 ± 0.03 | 0.62 ± 0.02 | 0.36 ± 0.02 |
| 48 | 0.44 ± 0.02 | 0.45 ± 0.03 | 0.47 ± 0.03 | 0.32 ± 0.01 |
| 72 | 0.61 ± 0.03 | 0.63 ± 0.03 | 0.62 ± 0.02 | 0.36 ± 0.02 |
| 96 | 0.61 ± 0.03 | 0.63 ± 0.03 | 0.62 ± 0.02 | 0.36 ± 0.02 |

Table 3. Absolute values for GSH [nmol/mg of protein] in NT2.A cultures following treatment with 20μM, 2μM, 0.2μM Aβ.

|  | Aβ1-42 concentration (μM) | | | |
| --- | --- | --- | --- | --- |
| Time (hours) | 20 | 2 | 0.2 | control |
| 6 | 1.79 ± 0.12 | 2.64 ± 0.06 | 2.68 ± 0.15 | 2.10 ± 0.04 |
| 24 | 2.20 ± 0.11 | 2.81 ± 0.13 | 2.46 ± 0.07 | 2.06 ± 0.06 |
| 48 | 2.12 ± 0.05 | 2.48 ± 0.09 | 2.85 ± 0.08 | 2.10 ± 0.07 |
| 72 | 1.86 ± 0.04 | 2.29 ± 0.03 | 2.27 ± 0.10 | 1.75 ± 0.07 |
| 96 | 1.67 ± 0.06 | 2.18 ± 0.07 | 2.37 ± 0.13 | 1.85 ± 0.12 |

Table 4. Absolute values for GSSG [nmol/mg of protein] in NT2.A cultures following treatment with 20μM, 2μM, 0.2μM Aβ.

|  | Aβ1-42 concentration (μM) | | | |
| --- | --- | --- | --- | --- |
| Time (hours) | 20 | 2 | 0.2 | control |
| 6 | 0.11 ± 0.00 | 0.11 ± 0.02 | 0.16 ± 0.02 | 0.10 ± 0.02 |
| 24 | 0.40 ± 0.04 | 0.40 ± 0.04 | 0.33 ± 0.02 | 0.22 ± 0.02 |
| 48 | 0.15 ± 0.02 | 0.16 ± 0.02 | 0.21 ± 0.02 | 0.10 ± 0.02 |
| 72 | 0.11 ± 0.03 | 0.10 ± 0.02 | 0.11 ± 0.02 | 0.10 ± 0.02 |
| 96 | 0.07 ± 0.01 | 0.08 ± 0.01 | 0.09 ± 0.02 | 0.11 ± 0.02 |

Table 5. Absolute values for NAD+ and NADH [pmol/mg of protein] in NT2.N/A cultures following treatment with 2μM Aβ.

|  | NAD+ | | NADH | |
| --- | --- | --- | --- | --- |
| Time (hours) | 2μM Aβ | control | 2μM Aβ | control |
| 6 | 686.63 ± 90.07 | 524.64 ± 73.28 | 2301.34 ± 37.02 | 2401.63 ± 88.63 |
| 24 | 1152.50 ± 78.19 | 919.67 ± 90.26 | 1273.57 ± 115.08 | 1390.84 ± 47.23 |
| 48 | 1545.43 ± 191.74 | 1602.81 ± 146.64 | 2300.04 ± 14.09 | 1690.88 ± 146.28 |
| 72 | 1321.92 ± 59.83 | 985.97 ± 38.83 | 2469.29 ± 174.20 | 1499.10 ± 128.06 |
| 96 | 347.50 ± 20.17 | 819.60 ± 17.49 | 1950.0 ± 21.20 | 1590.0 ± 107.73 |
